# The relationship between physical activity and depressive symptoms in college students: the sequential mediation effects of emotion regulation self-efficacy and sense of life meaning

**DOI:** 10.3389/fpsyg.2026.1824736

**Published:** 2026-05-22

**Authors:** Weiguo Chen, Haiyang Liu, Jiuyang Xu, Mingda Si, Xiaobin Zhong, Tianguo Li, Fengshu Zhu

**Affiliations:** 1 Department of Physical Education, Ningbo University of Technology, Ningbo, China; 2 Tongda College, Nanjing University of Posts and Telecommunications, Yangzhou, China; 3 Nanyang Technological University, Singapore, Singapore; 4 Nanchenji Middle School of Huai'an, Huai'an, China; 5 School of Physical Education, Yangzhou University, Yangzhou, China

**Keywords:** college students, depressive symptoms, emotion regulation self-efficacy, physical activity, sense of life meaning

## Abstract

**Objective:**

This research explores how emotion regulation self-efficacy and sense of life meaning affect the relationship between physical activity and depressive symptoms among college students.

**Methods:**

A total of 1,333 university students were evaluated with the Physical Activity Scale, Emotion Regulation Self-Efficacy Scale, Sense of Life Meaning Scale, and Depressive symptoms Scale. A sequential mediation model was established, and the mediating effects were examined via the bootstrap resampling approach.

**Results:**

(1) Physical activity was positively associated with both emotion regulation self-efficacy and sense of life meaning. A strong positive link emerged between emotion regulation self-efficacy and sense of life meaning. Physical activity was inversely associated with depressive symptoms, whereas emotion regulation self-efficacy and sense of life meaning similarly demonstrated inverse associations with depressive symptoms. (2) Emotion regulation self-efficacy and sense of life meaning serve as sequential mediators in the link between physical activity and depressive symptoms. The detailed mediation pathways are outlined below: Physical activity → Emotion regulation self-efficacy → Depressive symptoms (effect = −0.150, accounting for 20.41% of the total effect). Physical activity → sense of life meaning → Depressive symptoms (effect = −0.098, accounting for 13.33% of the total effect). Physical activity → Emotion regulation self-efficacy → sense of life meaning → Depressive symptoms (effect = −0.168, accounting for 22.86% of the total effect).

**Conclusion:**

(1) Physical activity is significantly and negatively associated with depressive symptoms among college students. (2) In college students, emotion regulation self-efficacy and sense of life meaning partially mediated the relationship between physical activity and depressive symptoms. (3) Emotion regulation self-efficacy and sense of life meaning together serve as sequential mediators linking physical activity to depressive symptoms among college students.

## 1 Research background

Depression has become one of the most prevalent mental health disorders worldwide (Fried, 2017). Depressive symptoms refer to a range of affective, cognitive, and behavioral manifestations associated with depression severity. They are commonly assessed in both clinical and non-clinical populations. Unlike clinically diagnosed major depression, depressive symptoms are conceptualized as a dimensional construct that varies in severity and does not necessarily meet diagnostic criteria. These symptoms typically include persistent sadness, reduced interest, feelings of worthlessness, and impaired concentration (Chen et al., 2022). Depressive symptoms are highly prevalent among college students and are linked to a range of adverse outcomes. Recent data indicate that 31.38% of Chinese college students have reported depressive symptoms, with this group also showing elevated risks of self-injurious behavior (Zhao et al., 2021; Wang et al., 2020). The COVID-19 pandemic has further increased the prevalence of depressive symptoms in this population (Jin et al., 2021). Elevated levels of depressive symptoms can impair academic performance and daily functioning, and in severe cases, may increase the risk of developing clinically significant depression. Therefore, investigating depressive symptoms among college students is important for early identification and prevention, as well as promoting mental and physical well-being.

Physical activity has been extensively acknowledged as a beneficial intervention for alleviating depressive symptoms, and it has been shown to be beneficial in reducing depressive symptoms and may serve as a complementary approach alongside pharmacological treatments (Carek et al., 2011). Evidence suggests that for adolescents with depression who show no improvement after 6 weeks of pharmacological treatment alone, integrating physical activity with pharmaceutical treatment results in a marked decrease in depressive manifestations (Tkachuk and Martin, 1999). This therapeutic effect is closely related to the multiple positive impacts of physical activity, such as enhancing positive emotions, reducing anxiety and depressive symptoms, increasing self-confidence, and improving cognitive function (Saeed et al., 2019). These benefits are attributed primarily to the promotion of endorphin production during exercise, since endorphins function as the body’s innate agents for pain relief and emotional regulation (Mikkelsen et al., 2017). A large-scale study involving over 40,000 university students revealed that those who engaged in regular aerobic exercise experienced significantly lower depressive symptoms and a reduction in suicidal behaviors (Taliaferro et al., 2009). Compared with other treatments, physical activity has notable advantages in alleviating depressive symptoms, including high compliance, minimal side effects, and stable efficacy, which are universally consistent across nations and cultures (Schuch et al., 2018). Moreover, substantial research has verified a notable association between physical activity and negative emotional states such as depression (Liu, 2024; Liu et al., 2021). Individuals who engage in high levels of physical activity are better at managing emotional fluctuations and reducing the likelihood of depressive symptoms than those with lower activity levels are (Zhang et al., 2021). Nevertheless, existing research has limitations, such as insufficient exploration of potential influencing factors between variables, weak explanations of underlying mechanisms, and a lack of sufficient studies in this area.

Emotional regulation self-efficacy is one’s belief in one’s capacity to effectively manage emotional reactions (Huang et al., 2015). Research indicates a strong link between emotion regulation self-efficacy and depressive symptoms (Tong et al., 2025; Bandura et al., 2001). Specifically, negative emotion regulation efficacy (e.g., regulating feelings of sadness or anger) directly predicts depressive symptoms 2 years later (Tamir and Mauss, 2009), whereas positive emotion regulation efficacy contributes to maintaining low levels of psychopathology and promotes positive psychological well-being (Lightsey and Talleyrand, 2013). Emotional regulation self-efficacy not only is a significant risk factor for depressive symptoms but also has an essential influence on emotional interactions and the shaping of self-perception. For example, when college students seek emotional interaction but encounter neglect or ineffective responses, the development of their positive self-schema may be hindered, leading to a decline in emotion regulation efficacy (Soffer et al., 2008). A strong sense of inefficacy, including emotion regulation inefficacy, is one of the core factors contributing to various forms of depressive symptoms (Tang et al., 2010). On the basis of the broaden-and-build theory put forward within the framework of positive emotions, experiencing positive emotions may widen an individual’s range of cognitive and behavioral responses, thereby fostering the accumulation of physical and psychological resources. In contrast, negative emotions hinder the building of such resources. When individuals excessively focus on negative emotions without effective release, depressive symptoms are more likely to occur (Russo-Netzer, 2018).

Drawing on Bandura’s social learning theory, strengthening one’s emotion regulation self-efficacy equips individuals to approach life difficulties with a more positive mindset, reducing anxiety and stress caused by a lack of confidence in problem solving and thereby promoting higher levels of mental health (Chen and Zheng, 2020). Engaging in physical activity can enhance emotion regulation self-efficacy by enabling individuals to gain a sense of achievement through exercise participation. Previous research has shown a strong connection between higher emotion regulation self-efficacy and greater physical activity among college students (Wu, 2017). A 10-week experimental intervention with college students revealed that moderate-to-high intensity physical activities most effectively enhanced emotion regulation self-efficacy (Liu et al., 2007). Additionally, students involved in group sports generally demonstrate higher levels of emotion regulation self-efficacy than do those participating in individual sports (Tamminen and Crocker, 2013). Longitudinal studies further indicate that physical activity not only strengthens college students’ emotion regulation self-efficacy and improves their social adaptability but also leads to significant improvements in positive emotions and state anxiety among students experiencing depressive symptoms after active exercise (Tang et al., 2022). Nevertheless, existing research has not provided sufficient evidence to elucidate the specific mechanisms through which physical activity alleviates depressive symptoms via emotion regulation self-efficacy.

The sense of life meaning refers to an individual’s understanding of their purpose in life, guided by meaningful goals, and the perception that their life holds significance (George, 2016). In recent years, the sense of meaning of life has been increasingly recognized as a key area of focus within mental health research. Studies have revealed that a deficiency in life meaning is associated with an increased risk of unfavorable outcomes, including psychological disorders, physical impairment, and suicide (He et al., 2023). This issue is particularly prominent among college students, who face increasing academic pressure and life challenges. A sense of purposelessness can impair mental well-being and disrupt academic and social adjustment (Ge et al., 2021). The theory of deficit restoration posits that individuals experiencing adversity often initiate a process of rediscovering life meaning, adjusting critical aspects of their lives, and reconstructing meaning frameworks to redefine their sense of self-worth (Li et al., 2018). Research has shown that physical activity notably predicts an individual’s sense of meaning in life (Ding et al., 2016; Ju et al., 2017). Specifically, physical activity enhances the sense of meaning by increasing peer interaction frequency, promoting interpersonal communication, and creating a positive exercise environment. These factors enable college students to experience pleasant emotions, exhibit more prosocial behaviors, strengthen their perception of life meaning and lower the likelihood of experiencing a depressive symptoms (Demir-Kassem et al., 2025). Furthermore, research involving participants experiencing depressive symptoms has indicated that targeted interventions to increase their sense of life effectively assist individuals in emotional regulation, increasing positive emotions, and increasing overall quality of life (Xu et al., 2020). Emotional regulation self-efficacy, regarded as an essential psychological asset, has been shown to significantly influence the fostering of one’s sense of meaning in life (Meng et al., 2024). Individuals with higher emotion regulation self-efficacy are better equipped to adapt to social situations, demonstrate higher confidence levels, effectively solve life problems, and experience a greater sense of life meaning (Webb et al., 2012). Conversely, individuals lacking emotion regulation self-efficacy may struggle to cope with adverse life events, leading to chaotic personal lives, a diminished sense of meaning, and a greater likelihood of mental health problems such as depression and anxiety (Benight and Bandura, 2004). Despite these findings, current research has focused primarily on the separate effects of emotion regulation self-efficacy or sense of life meaning on depressive symptoms. However, the mediating role of these factors in relation to depressive symptoms remains unclear.

Although previous studies have examined the relationships among physical activity, emotion regulation self-efficacy, sense of life meaning, and depressive symptoms, these variables have largely been investigated in isolation. Existing research has primarily focused on pairwise associations, with limited attention to how they operate within an integrated psychological framework, leaving the mechanisms linking behavioral engagement to mental health outcomes insufficiently clarified. From a psychological resource perspective, physical activity may enhance individuals’ perceived capacity to regulate emotions, as reflected in increased emotion regulation self-efficacy. This improvement in self-regulatory capacity may further facilitate the development of a coherent and meaningful life framework, thereby strengthening individuals’ sense of life meaning. In this process, emotion regulation self-efficacy may function as a proximal psychological resource, whereas sense of life meaning represents a more distal, meaning-oriented outcome.

Therefore, rather than operating as independent parallel mediators, these variables may form a sequential psychological pathway through which physical activity is associated with depressive symptoms. Examining such a sequential mediation model may provide a more nuanced understanding of the mechanisms underlying this relationship. At the same time, alternative explanations should be considered. For example, sense of life meaning may also influence emotion regulation processes, or these variables may operate independently in parallel. Testing a sequential mediation model may therefore help clarify these competing possibilities and advance understanding of the psychological pathways linking physical activity and depressive symptoms. To better understand the relationships among these variables and clarify the underlying mechanisms, the following hypotheses are proposed.

*H1*: Among university students, higher levels of physical activity are inversely associated with depressive symptoms.

*H2*: Emotion regulation self-efficacy mediates the link between physical activity and depressive symptoms among university students.

*H3*: Sense of life meaning serves as a mediator in the relationship between physical activity and depressive symptoms among university students.

*H4*: Emotion regulation self-efficacy and sense of life meaning may sequentially mediate the association between physical activity and depressive symptoms among university students.

## 2 Research methods

### 2.1 Research participants

Employing a random sampling approach, university students from selected institutions in Zhejiang and Jiangsu Provinces in China were recruited as participants. The questionnaires were distributed via the Wenjuan Xing app. Before the survey, the participants received an explanation outlining the study’s objectives and participation guidelines, with an emphasis on the anonymity and confidentiality of the questionnaire. The Ethics Committee of Yangzhou University approved this study (YZU-EC-2025-045). A total of 1,359 questionnaires were administered. Following the removal of 26 invalid responses due to uniform response patterns, 1,333 valid responses were collected, resulting in a 98.08% response rate. Among the respondents, there were 654 male and 679 female university students.

### 2.2 Assessment instruments

#### 2.2.1 Physical activity rating scale

The scale was developed and revised by Liang (1994) and assesses physical activity in three domains: intensity, duration, and frequency. The formula used for calculation was as follows: Physical Activity = Exercise Intensity × (Exercise Duration – 1) × Exercise Frequency. Each of the three dimensions is rated on a 5-point Likert scale. On the basis of the total score, the participants were categorized into three activity levels: low (≤19), moderate (20–42), and high (≥43). The scale had a Cronbach’s alpha of 0.732, suggesting good internal consistency in this research.

#### 2.2.2 Emotion regulation self-efficacy scale

The scale was adapted by Wen et al. (2009) and contains 12 items covering three dimensions: confidence in expressing positive emotions, managing depressive or distressed emotions, and regulating anger-related emotional states. Each item was evaluated via a 5-point Likert scale, with higher scores reflecting stronger perceived self-efficacy in emotion regulation. In this research, the scale had a Cronbach’s alpha of 0.925, suggesting outstanding internal consistency.

#### 2.2.3 Sense of life meaning scale

Liu and Gan (2010) localized and revised the scale, which consists of 9 items that evaluate two core aspects: the existence of meaning and the pursuit of meaning. The responses were scored on a 7-point Likert scale, with higher scores signifying a stronger sense of life meaning. The Cronbach’s alpha for this scale in the present sample was 0.840.

#### 2.2.4 Depressive symptoms scale

Depressive symptoms were assessed using the Patient Health Questionnaire-9 (PHQ-9; Kroenke et al., 2001), a widely used self-report instrument designed to measure the severity of depressive symptomatology in both clinical and non-clinical populations. Including diminished interest, persistent low mood, sleep problems, fatigue, appetite disturbances, feelings of worthlessness, impaired concentration, psychomotor slowing, and suicidal thoughts. The scale is made up of 9 items, each of which is scored on a 4-point Likert scale ranging from 0 to 3. Higher total scores indicate more severe depressive symptoms. In this research, the internal consistency of the scale was verified, with a Cronbach’s alpha value of 0.817.

### 2.3 Confirmatory factor analysis

To examine the construct validity of the multi-item scales, a confirmatory factor analysis was conducted using the lavaan package in R. Physical activity was operationalized as a composite index based on intensity, duration, and frequency, and was therefore treated as an observed variable rather than a latent construct in the CFA. The three-factor measurement model demonstrated a marginally acceptable fit to the data, *χ*^2^(397) = 2301.51, *P* < 0.001, CFI = 0.892, TLI = 0.881, RMSEA = 0.060, 90% CI [0.058, 0.062], and SRMR = 0.049. Although the CFI and TLI were slightly below the conventional cutoff of 0.90, the overall pattern of fit indices, particularly the RMSEA and SRMR, suggested an acceptable level of approximate model fit. Given the relatively large sample size, the significant chi-square statistic was expected.

As shown in Table 1, the standardized factor loadings were generally acceptable. The loadings ranged from 0.660 to 0.763 for emotional regulation self-efficacy, from 0.487 to 0.642 for depressive symptoms, and from 0.085 to 0.741 for meaning in life. Although SLM2 showed a relatively weak standardized factor loading, it was retained because it is conceptually consistent with the other meaning in life items and reflects a similar content domain of the target construct. Retaining this item also helped preserve the original scale structure and maintain consistency with subsequent analyses. Overall, these findings suggest that the measurement model was acceptable and provided reasonable evidence for the construct validity of the key study variables.

**Table 1:** Standardized factor loadings for the measurement model.

Construct	Item	Standardized loading
Emotion regulation self-efficacy	ERSE1	0.660
	ERSE2	0.679
	ERSE3	0.676
	ERSE4	0.660
	ERSE5	0.763
	ERSE6	0.695
	ERSE7	0.717
	ERSE8	0.739
	ERSE9	0.724
	ERSE10	0.753
	ERSE11	0.721
	ERSE12	0.747
Sense of life meaning	SLM1	0.678
	SLM2	0.085
	SLM3	0.670
	SLM4	0.720
	SLM5	0.735
	SLM6	0.686
	SLM7	0.743
	SLM8	0.723
	SLM9	0.695
Depressive symptoms	PHQ1	0.487
	PHQ2	0.604
	PHQ3	0.582
	PHQ4	0.573
	PHQ5	0.548
	PHQ6	0.642
	PHQ7	0.581
	PHQ8	0.598
	PHQ9	0.541

All factor loadings are standardized and statistically significant.

### 2.4 Statistical processing

SPSS 27.0 was used to conduct the statistical analyses. Harman’s single-factor test was used to assess common method bias. Pearson correlation analysis was employed to examine the relationships between variables. The sequential mediation model was constructed via Process macro4.0, and the mediating effects were examined via path analysis combined with the bootstrap resampling technique. A mediation effect was deemed statistically significant when its 95% CI did not include zero.

## 3 Research results

### 3.1 Common method bias test

To evaluate possible common method bias, we conducted Harman’s single-factor test on the entire dataset (Zhou and Long, 2004). The unrotated exploratory factor analysis revealed five factors with eigenvalues greater than 1, with the main factor accounting for 28.29% of the variance, significantly below the 40% criterion. Therefore, this study does not have an issue of common method bias.

### 3.2 Descriptive statistics and correlation analysis

To examine the relationships among the variables, Pearson’s correlation analysis was employed. As presented in Table 2, positive correlations among physical activity, emotion regulation self-efficacy, and sense of life meaning. Emotion regulation self-efficacy was positively correlated with sense of life meaning. In addition, physical activity was negatively correlated with depressive symptoms. Both emotion regulation self-efficacy and sense of life meaning were negatively correlated with depressive symptoms.

**Table 2:** Descriptive statistics and correlation matrix of the variables.

Variables	*M* ± SD	1	2	3	4
Physical activity	29.24 ± 17.88	1			
Emotional regulation self-efficacy	39.65 ± 9.51	0.34^**^	1		
Sense of life meaning	39.44 ± 9.76	0.24^**^	0.49^**^	1	
Depressive symptoms	6.43 ± 4.28	−0.17^**^	−0.25^**^	−0.31^**^	1

**p < 0.01.

### 3.3 Sequence mediation effect test

Prior to analysis, the independent and mediating variables were standardized. Mediation was assessed using Hayes’ PROCESS macro (Model 6). The regression analysis results (see Table 3) indicated that physical activity was significantly positively associated with emotion regulation self-efficacy (*β* = 0.34, *t* = 13.28, *p* < 0.001) and sense of life meaning (*β* = 0.09, *t* = 3.61, *p* < 0.001), indicating that higher levels of physical activity are associated with higher levels of emotion regulation self-efficacy and sense of life meaning. Emotion regulation self-efficacy was also significantly positively associated with sense of life meaning (*β* = 0.45, *t* = 18.10, *p* < 0.001), indicating that higher emotion regulation self-efficacy is associated with a stronger sense of life meaning. In addition, physical activity (*β* = −0.31, *t* = −2.70, *p* < 0.001), emotion regulation self-efficacy (*β* = −0.43, *t* = −3.33, *p* < 0.001), and sense of life meaning (*β* = −1.07, *t* = −8.43, *p* < 0.001) were significantly negatively associated with depressive symptoms, indicating that higher levels of these variables are associated with lower levels of depressive symptoms.

**Table 3:** Sequence mediation model test.

Regression equation		Overall fit indices			Significance Level	
Outcome variable	Predictor variable	*R*	*R^2^*	*F*	*β*	*t*
Depressive symptoms	Physical activity	0.172	0.029	40.37^***^	−0.735	−6.35^***^
Emotional regulation self-efficacy	Physical activity	0.342	0.117	176.60^***^	0.342	13.28^***^
Sense of life meaning	Physical activity	0.497	0.247	218.30^***^	0.091	3.61^***^
	Emotional regulation self-efficacy				0.458	18.10^***^
Depressive symptoms	Physical activity	0.344	0.118	59.40^***^	−0.319	−2.70^***^
	Emotional regulation self-efficacy				−0.438	−3.33^***^
	Sense of life meaning				−1.073	−8.43^***^

***p < 0.001.

**Figure 1:**
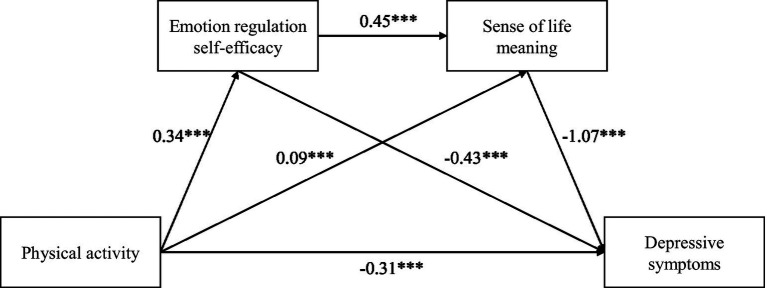
Sequence mediation model diagram. ***P < 0.001.

To further examine the mediating effects, a bias-corrected bootstrap procedure with 5,000 resamples was used to estimate 95% confidence intervals (CI). An effect was considered statistically significant when the 95% CI did not include zero. As shown in Table 4, physical activity showed a significant direct association with depressive symptoms (effect = −0.319, accounting for 43.40%). Partial mediation effects were also significant (as shown in Figure 1). Ind 1: Physical activity → emotional regulation self-efficacy → depressive symptoms (effect = −0.150, accounting for 20.41%); Ind 2: Physical activity → sense of life meaning → depressive symptoms (effect = −0.098, accounting for 13.33%); Ind 3: Physical activity → emotional regulation self-efficacy → sense of life meaning → depressive symptoms (effect = −0.168, accounting for 22.86%).

**Table 4:** Bootstrap method mediation effect test.

Path	Effect	Boot SE	Boot LLCI	Boot ULCI	Percentage
Total effect	−0.735	0.116	−0.962	−0.508	100.00%
Direct effect	−0.319	0.118	−0.551	−0.087	43.40%
Total indirect effect	−0.416	0.062	−0.543	−0.304	56.60%
Ind 1	−0.150	0.044	−0.239	−0.066	20.41%
Ind 2	−0.098	0.031	−0.164	−0.042	13.33%
Ind 3	−0.168	0.028	−0.227	−0.118	22.86%

## 4 Discussion

This study developed a sequential mediation framework to investigate how physical activity is related to depressive symptoms, with a specific focus on the mediating roles of emotion regulation self-efficacy and sense of life meaning. The findings demonstrated that increased physical activity was correlated with lower levels of depressive symptoms, with emotion regulation self-efficacy and sense of life meaning acting as sequential mediators in this relationship. These findings offer empirical evidence for the early identification and prevention of depressive symptoms among university students.

### 4.1 Relationships between physical activity and depressive symptoms

Studies indicate that college students who engage in regular exercise tend to experience fewer symptoms of depression, suggesting that staying active may help ward off low moods. The more physically active students are, the less likely they are to struggle with depressive symptoms. However, the magnitude of this association was relatively small, and physical activity may be only one of multiple factors related to depressive symptoms. These findings supported Hypothesis 1 and aligned with previous empirical research (Xiao et al., 2017). Physical activity helps alleviate depressive symptoms through a variety of physiological and psychological mechanisms. Physiologically, physical activity can regulate neurotransmitter levels (such as serotonin, dopamine, and endorphins) while reducing the secretion of stress hormones such as cortisol, improving an individual’s emotional state (Wang et al., 2021). Psychologically, physical activity enhances self-efficacy, increases individual confidence, and shapes positive self-perception, effectively reducing feelings of helplessness and low self-esteem (Wang, 2021). Additionally, physical activity provides a channel for emotional expression, which contributes to the mitigation of negative emotional build-up, lowering the likelihood of the onset and worsening of depressive symptoms. However, it is also plausible that depressive symptoms may reduce physical activity levels, limiting opportunities to regulate emotions. According to the transactional model of stress and coping (Lazarus and Folkman, 1984), stress may weaken an individual’s ability to engage in coping mechanisms, such as physical activity, which might otherwise help regulate emotions. In this case, depressive symptoms could limit energy and motivation, thereby reducing physical activity and disrupting the emotional regulation process, the interplay between physical activity and depressive symptoms is likely complex, with each factor influencing the other in a dynamic and reciprocal manner.

### 4.2 Mediating role of emotional regulation self-efficacy

Research has indicated that emotion regulation self-efficacy mediates the connection between physical activity and depressive symptoms. Physical activity not only improves emotion regulation self-efficacy but also reduces the risk of depressive symptoms among university students. This result aligns with those of prior studies and further supports the validity of Hypothesis 2. Research has shown that improving emotion regulation self-efficacy may represent a potential pathway through which exercise interventions alleviate depressive symptoms (Noetel et al., 2024). This is particularly true for individuals who are experiencing emotional lows or facing a greater risk of depression. Through physical activity, these individuals can enhance their emotion regulation self-efficacy, enabling them to cope with and manage depressive emotions more effectively (Mu et al., 2024). Self-determination theory emphasizes that when individuals with depressive symptoms experience increased competence and autonomy through engaging in physical activity, their confidence and ability in emotion regulation are significantly enhanced, thereby allowing them to effectively regulate negative emotions (Ryan and Deci, 2000). Consequently, regular physical activity helps boost college students’ self-efficacy in managing emotions, thereby mobilizing more psychological resources to identify and regulate emotional states, express positive emotions, and facilitate better social adaptation and interpersonal interactions. These changes contribute to a reduction in the occurrence of depressive symptoms.

### 4.3 Mediating role of the sense of life meaning

Research has shown that sense of life meaning acts as a mediator in the connection between physical activity and depressive symptoms. Specifically, physical activity is associated with depressive symptoms both directly and indirectly through an individual’s sense of meaning in life, which may be associated with lower depressive symptoms. This finding supports Hypothesis 3. Previous studies have identified the absence of meaning in life as one of the key factors contributing to depressive symptoms (Boreham and Schutte, 2023). As an effective means of enhancing life meaning, physical activity helps individuals achieve self-worth, set goals, and overcome challenges, thus effectively alleviating depressive symptoms (Liu et al., 2024). This conclusion reflects the analytical results reached in this study. Existential theory suggests that the formation of life meaning is not only the result of internal exploration but also heavily influenced by the context and environment in which an individual finds themselves (Steger and Kashdan, 2009). Physical activity offers college students an experiential context that facilitates self-actualization, where individuals, by facing challenges and continuously surpassing themselves, can discover and construct their life meaning within specific contexts, thereby positively impacting their mental health. From a psychological perspective, the enhancement of life meaning allows individuals to better find direction and purpose in life, and this goal-oriented behavior helps combat the negative effects of depressive symptoms (Zhang et al., 2018). As life meaning increases, individuals’ sense of life identity and belonging strengthens, thereby boosting their resilience against daily life pressures and hurdles.

### 4.4 Sequential mediation of emotion regulation self-efficacy and sense of life meaning

Research has indicated that emotion regulation self-efficacy and sense of life meaning sequentially mediate the association between physical activity and depressive symptoms. Hypothesis 4 was supported, further verifying the strong link between emotion regulation self-efficacy and sense of life meaning. People with elevated emotion regulation self-efficacy tend to experience a greater frequency of positive emotional states, which in turn reinforce their perception of life meaning, forming a positive feedback loop. This process can be understood via Fredrickson (2001) broaden-and-build framework for positive emotions: Experiencing positive emotions leads to expanded attentional scope, greater cognitive flexibility, and enhanced psychological resilience. This state enables them to adapt more effectively when facing stress or adversity. Moreover, emotion regulation self-efficacy may represent a potential pathway linking physical activity to depressive symptoms. Physical activity helps improve individuals’ physiological state and enhances social interactions, thereby increasing their confidence in managing negative emotions (Sun et al., 2023), enabling individuals to better manage emotional fluctuations and reduce the accumulation of unpleasant emotional experiences. The sense of life meaning, as a continuation effect of emotional regulation self-efficacy, has been fully validated in this study for its buffering role against depressive symptoms. After emotional regulation through physical activity is enhanced, individuals are more likely to discover positive meaning and goals in life. The increased sense of meaning of life makes them more positive when facing setbacks and challenges, thereby reducing the occurrence of depressive symptoms (Yang, 2024).

While this study supports the buffering role of emotion regulation self-efficacy and sense of life meaning in mitigating depressive symptoms, the relationship between these variables and depressive symptoms is complex and not entirely linear. Emotion regulation self-efficacy is influenced by both internal factors, such as personality traits, and external factors like stress and social support (Doménech et al., 2024). Even individuals with high self-efficacy may struggle under chronic stress, limiting its protective effect. Physical activity, which serves as a mediator in this model, enhances emotional well-being, but its impact on depressive symptoms can vary across individuals. Barriers such as limited access to exercise or physical constraints may reduce its potential effectiveness, highlighting the importance of considering individual differences (Bauman et al., 2012). Similarly, sense of life meaning, though associated with better mental health, is a dynamic and subjective construct. It can fluctuate depending on life experiences and disruptions, with some individuals facing challenges in maintaining meaning in the face of adversity (Steger et al., 2008). Taken together, these findings underscore the intricate interplay among physical activity, emotion regulation self-efficacy, sense of life meaning, and depressive symptoms, pointing to the importance of an integrated approach that recognizes the multidimensional nature of mental health and emotional resilience.

### 4.5 Research value and limitations

This study identified a direct association between physical activity and a reduction in depressive symptoms in college students and explored how this relationship is indirectly influenced by improved emotion regulation self-efficacy and an enhanced sense of life meaning. This discovery enhances our theoretical comprehension of how physical activity impacts mental health, emphasizing its potential to improve emotional regulation and reduce symptoms of mental distress. While earlier research has largely emphasized how physical activity directly influences physical well-being and emotional states, this study focused on the mediating roles of emotion regulation self-efficacy and sense of life meaning, offering a novel perspective for psychological theory.

Therefore, universities should consider integrating physical activity into their psychological health education systems and regularly organizing diverse physical activities, although the effectiveness of such practices needs to be further confirmed through longitudinal and experimental research. By diversifying the forms of physical activity, such as group sports, outdoor challenges, and campus competitions, students’ participation interests may be enhanced. Additionally, these activities can improve students’ self-efficacy and emotional regulation skills, thereby effectively reducing psychological stress, relieving negative emotions and facilitating the holistic development of individuals’ overall physical and psychological well-being.

Despite offering valuable insights, this study has several limitations. First, the cross-sectional design used in this research, coupled with the absence of long-term follow-up data, precludes the ability to rule out potential reverse causality. Future research should adopt longitudinal or experimental designs to evaluate the lasting effects of physical activity on depressive symptoms. Secondly, all variables were assessed using self-report measures, which are susceptible to response biases. While Harman’s single-factor test was conducted to examine common method bias, this approach has limited sensitivity, meaning the possibility of common method variance cannot be fully excluded. Future studies should employ more rigorous statistical techniques, such as latent method factor models, or utilize multi-source and multi-method data to reduce potential bias. Moreover, the sample in this study primarily consisted of college students, which limits the generalizability of the findings. Emotional regulation and the perception of life meaning may differ across individuals from various age groups, sociocultural contexts, and demographics. As such, future research should aim to include a more diverse sample to enhance the generalizability of the results.

## 5 Conclusion

This study revealed that physical activity was significantly negatively associated with depressive symptoms among university students. Emotional regulation self-efficacy and sense of life meaning partially mediate this effect. Moreover, these factors sequentially influence the relationship between physical activity and depressive symptoms, emphasizing their vital role in reducing depression.

## Data Availability

The raw data supporting the conclusions of this article will be made available by the authors, without undue reservation.

## References

[ref1] Bandura A. Barbaranelli C. Caprara G. V. Pastorelli C. (2001). Self-efficacy beliefs as shapers of children's aspirations and career trajectories. Child Dev. 72, 187–206. doi: 10.1111/1467-8624.00273, 11280478

[ref2] Bauman A. E. Reis R. S. Sallis J. F. Wells J. C. Loos R. J. Martin B. W. . (2012). Correlates of physical activity: why are some people physically active and others not? Lancet 380, 258–271. doi: 10.1016/S0140-6736(12)60735-1, 22818938

[ref3] Benight C. C. Bandura A. (2004). Social cognitive theory of post-traumatic recovery: the role of perceived self-efficacy. Behav. Res. Ther. 42, 1129–1148. doi: 10.1016/j.brat.2003.08.008, 15350854

[ref4] Boreham I. D. Schutte N. S. (2023). The relationship between purpose in life and depression and anxiety: a meta-analysis. J. Clin. Psychol. 79, 2736–2767. doi: 10.1002/jclp.23576, 37572371

[ref5] Carek P. J. Laibstain S. E. Carek S. M. (2011). Exercise for the treatment of depression and anxiety. Int. J. Psychiatry Med. 41, 15–28. doi: 10.2190/PM.41.1.c, 21495519

[ref6] Chen F. Lian J. Zhang G. Guo C. (2022). Semantics–prosody Stroop effect on English emotion word processing in Chinese college students with trait depression. Front. Psych. 13:889476. doi: 10.3389/fpsyt.2022.889476, 35733799 PMC9207235

[ref7] Chen B. Z. Zheng X. (2020). The positive self-presentation on social media and life satisfaction: the mediating roles of self-efficacy and emotional intelligence. J. Nanjing Univ. Chin. Med. 21, 53–57. doi: 10.20060/j.cnki.issn1009-3222.2020.01.011

[ref8] Demir-Kassem S. Frey A.-L. McCabe C. (2025). Meaning in life mediates the effects of sense of self and prosocial behaviours on anhedonia: a path analysis. J. Affect. Disord. 368, 503–512. doi: 10.1016/j.jad.2024.09.106, 39303888

[ref9] Ding S. Y. Zhang W. B. Liu T. T. (2016). The impact of physical activity on the sense of life meaning among university students. Acta Psychol. Sin. 48, 479–489. doi: 10.16835/j.cnki.1000-9817.2016.03.037

[ref10] Doménech P. Tur-Porcar A. M. Mestre-Escrivá V. (2024). Emotion regulation and self-efficacy: the mediating role of emotional stability and extraversion in adolescence. Behav. Sci. 14:206. doi: 10.3390/bs14030206, 38540509 PMC10968139

[ref11] Fredrickson B. L. (2001). The role of positive emotions in positive psychology: the broaden-and-build theory of positive emotions. Am. Psychol. 56, 218–226. doi: 10.1037/0003-066X.56.3.218, 11315248 PMC3122271

[ref12] Fried E. I. (2017). The 52 symptoms of major depression: lack of content overlap among seven common depression scales. J. Affect. Disord. 208, 191–197. doi: 10.1016/j.jad.2016.10.019, 27792962

[ref13] Ge Y. Zhang X. Cheng Y. Zhang H. (2021). Associated effects of meaning in life and social adjustment in undergraduate students after COVID-19. Front. Psych. 12:771082. doi: 10.3389/fpsyt.2021.771082, 34925099 PMC8682051

[ref14] George L. K. (2016). The concept of meaning in life: an overview. J. Soc. Issues 72, 164–176. doi: 10.1111/josi.12163

[ref15] He X.-X. Wang X.-Q. Steger M. F. Ji L.-J. Jing K. Liu M.-F. . (2023). Meaning in life and psychological distress: a meta-analysis. J. Res. Pers. 104:104381. doi: 10.1016/j.jrp.2023.104381

[ref16] Huang S. H. Cai F. X. Liu P. L. Zhang W. Gong W. J. (2015). Parent–child relationship and school adaptation in middle school students: the mediating role of emotional regulation self-efficacy. Chin. J. Clin. Psychol. 23, 171–173, 177. doi: 10.16128/j.cnki.1005-3611.2015.01.039

[ref17] Jin Y. L. Chang W. W. Chang X. Zhu L. J. Fang Z. M. Chen Y. . (2021). Analysis of the psychological health and influencing factors of university students during online learning amid the COVID-19 pandemic. Chin. J. Sch. Health 42, 574–578. doi: 10.16835/j.cnki.1000-9817.2021.04.022

[ref18] Ju H. Sar A. H. Park C. (2017). The relationship between physical activity, meaning in life, and subjective vitality in community-dwelling older adults. Arch. Gerontol. Geriatr. 73, 120–124. doi: 10.1016/j.archger.2017.08.001, 28802214

[ref19] Kroenke K. Spitzer R. L. Williams J. B. (2001). The PHQ-9: validity of a brief depression severity measure. J. Gen. Intern. Med. 16, 606–613. doi: 10.1046/j.1525-1497.2001.016009606.x, 11556941 PMC1495268

[ref20] Lazarus R. S. Folkman S. (1984). Stress, Appraisal, and Coping. New York: Springer Publishing Company.

[ref21] Li Z. H. Zhao M. J. Liu H. Y. Liu Y. N. Peng K. P. (2018). The reasons for seeking meaning in life: growth or crisis. Adv. Psychol. Sci. 26, 2192–2203. doi: 10.3724/SP.J.1042.2018.02192

[ref22] Liang D. Q. (1994). Stress levels of university students and their relationship with physical exercise. Chin. J. Mental Health 8, 5–6. doi: 10.3321/j.issn:1000-6729.1994.01.003

[ref23] Lightsey O. R. Talleyrand R. M. (2013). Emotion regulation self-efficacy and psychological distress: the role of self-efficacy for regulating negative and positive emotions. J. Appl. Soc. Psychol. 43, 837–846.

[ref24] Liu Y. (2024). The effects of physical exercise on the sense of life meaning among university students: the chain mediating effect of personality traits and emotional intelligence. Psychol. Res. 4, 360–366.

[ref25] Liu S. S. Gan Y. Q. (2010). The reliability and validity of the Chinese version of the meaning in life questionnaire among university students. Chin. J. Mental Health 24, 478–482. doi: 10.3969/j.issn.1000-6729.2010.06.021

[ref26] Liu J. K. He X. B. Zhang Y. L. (2021). Physical exercise, parent–child relationship, and adolescent mental health: evidence from the China education tracking survey. Chin. Youth Stud. 42, 103–112.

[ref27] Liu H. Y. Tong Z. G. Yan J. (2007). The effects of different time durations and intensities of aerobics exercise on self-efficacy and mental health in female college students. J. Xi'an Phys. Educ. Univ. 24, 125–129. doi: 10.3969/j.issn.1001-747X.2007.01.034

[ref28] Liu S. Zhou X. Li Z. H. (2024). The impact of physical activity on adolescent health: the chain mediating role of academic stress and sleep quality. China Sport Sci. Technol. 60, 55–60.

[ref29] Meng S. Bai B. Bai C. Shrestha S. Ren Y. (2024). Invalidating environment and meaning in life: the chain mediating effects of regulatory emotional self-efficacy and basic psychological needs satisfaction. Child Abuse Negl. 151:106736. doi: 10.1016/j.chiabu.2024.106736, 38522146

[ref30] Mikkelsen K. Stojanovska L. Polenakovic M. Bosevski M. Apostolopoulos V. (2017). Exercise and mental health. Maturitas 106, 48–56. doi: 10.1016/j.maturitas.2017.09.003, 29150166

[ref31] Mu F.-z. Liu J. Lou H. Zhu W.-d. Wang Z.-c. Li B. (2024). How breaking a sweat affects mood: the mediating role of self-efficacy between physical exercise and emotion regulation ability. PLoS One 19:e0303694. doi: 10.1371/journal.pone.0303694, 38870188 PMC11175485

[ref32] Noetel M. Sanders T. Gallardo-Gómez D. Taylor P. del Pozo Cruz B. van den Hoek D. . (2024). Effect of exercise for depression: systematic review and network meta-analysis of randomised controlled trials. BMJ 384:e075847. doi: 10.1136/bmj-2023-075847, 38355154 PMC10870815

[ref33] Russo-Netzer P. (2018). Meaning in life and mental health: the role of meaning in buffering against depression and enhancing emotional well-being. J. Clin. Psychol. 74, 1833–1843. doi: 10.1002/jclp.22658, 29696640

[ref34] Ryan R. M. Deci E. L. (2000). Self-determination theory and the facilitation of intrinsic motivation, social development, and well-being. Am. Psychol. 55, 68–78. doi: 10.1037/0003-066X.55.1.68, 11392867

[ref35] Saeed S. A. Cunningham K. Bloch R. M. (2019). Depression and anxiety disorders: benefits of exercise, yoga, and meditation. Am. Fam. Physician 99, 620–627. 31083878

[ref36] Schuch F. B. Vancampfort D. Firth J. Rosenbaum S. Ward P. B. Silva E. S. . (2018). Physical activity and incident depression: a meta-analysis of prospective cohort studies. Am. J. Psychiatry 175, 631–648. doi: 10.1176/appi.ajp.2018.17111194, 29690792

[ref37] Soffer D. Glick A. Kivenson T. (2008). Self-efficacy and depression in college students: the role of emotional interactions. J. Coll. Stud. Dev. 49, 147–160.

[ref38] Steger M. F. Kashdan T. B. (2009). Depression and everyday social activity, belonging, and well-being. J. Couns. Psychol. 56, 289–300. doi: 10.1037/a0015416, 20428460 PMC2860146

[ref39] Steger M. F. Kashdan T. B. Sullivan B. A. Lorentz D. (2008). Understanding the search for meaning in life: personality, cognitive style, and the dynamic between seeking and experiencing meaning. J. Pers. 76, 199–228. doi: 10.1111/j.1467-6494.2007.00484.x, 18331281

[ref40] Sun W. X. Wang X. Yu M. X. Zhao Q. Y. Zhou X. J. (2023). The level of physical activity participation and depression symptoms among university students: the mediating role of social support. Chin. J. Health Stat. 40, 421–424, 428.

[ref41] Taliaferro L. A. Rienzo B. A. Pigg R. M. Miller M. D. Dodd V. J. (2009). Associations between physical activity and reduced rates of hopelessness, depression, and suicidal behavior among college students. J. Am. Coll. Heal. 57, 427–436. doi: 10.3200/JACH.57.4.427-436, 19114382

[ref42] Tamir M. Mauss A. S. (2009). Prejudice and emotion regulation: the role of cognitive control in the regulation of negative emotions. Emotion 9, 379–388.

[ref43] Tamminen K. A. Crocker P. R. E. (2013). “I control my own emotions for the sake of the team”: emotional self-regulation and interpersonal emotion regulation among female high-performance curlers. Psychol. Sport Exerc. 14, 737–747. doi: 10.1016/j.psychsport.2013.05.002, 38826717

[ref44] Tang S. Chen H. Wang L. Lu T. Yan J. (2022). The relationship between physical exercise and negative emotions in college students in the post-epidemic era: the mediating role of emotion regulation self-efficacy. Int. J. Environ. Res. Public Health 19:12166. doi: 10.3390/ijerph191912166, 36231469 PMC9566100

[ref45] Tang D. L. Dong Y. Yu G. L. Wen S. F. (2010). Emotional regulation self-efficacy: a new research topic. Adv. Psychol. Sci. 18, 598–604.

[ref46] Tkachuk G. A. Martin G. L. (1999). Exercise therapy for patients with psychiatric disorders: research and clinical implications. Prof. Psychol. Res. Pract. 30, 275–282. doi: 10.1037/0735-7028.30.3.275

[ref47] Tong J. Xue M. Gao R. Wang W. Li H. (2025). Interpersonal sensitivity, emotion regulation self-efficacy, and depression symptoms in medical students: a network analysis. J. Xuzhou Med. Univ. 45, 332–338. doi: 10.12467/j.issn.2096-3882.20250336

[ref48] Wang M. Y. (2021). The impact of adolescent physical activity on depressive tendencies: the mediating effects of motivation and subjective experience. Sports Sci. 41, 78–85, 110.

[ref49] Wang M. Y. Han F. F. Liu J. Huang K. L. Peng H. Y. Huang M. T. . (2020). A meta-analysis of the detection rate and related factors of depression symptoms in university students. Chin. J. Ment. Health 34, 1041–1047.

[ref50] Wang Y. J. Lin J. Y. Xie T. E M. Wu Q. Lei Y. (2021). How physical exercise affects adolescent depression: a review and outlook. Psychol. Sci. 44, 1208–1215.

[ref51] Webb T. L. Miles E. Sheeran P. (2012). Dealing with feeling: a meta-analysis of the effectiveness of strategies derived from the process model of emotion regulation. Psychol. Bull. 138, 775–808. doi: 10.1037/a0027600, 22582737

[ref52] Wen S. F. Tang D. L. Yu G. L. (2009). Applied research on emotional regulation self-efficacy. Psychol. Sci. 32, 666–668.

[ref53] Wu Y. C. (2017) An Experimental Study on the Effects of Basketball and Jump Rope Exercise on Self-Efficacy and Mood State in College Students [Master's thesis, Yangzhou University]

[ref54] Xiao R. Wang Q. X. Zheng Z. S. (2017). The relationship between physical activity and depression in university students: the influence of gender differences. Chin. J. Sports Med. 36, 812–816, 822.

[ref55] Xu L. X. Shi Y. S. Xiong C. E. Liu Z. S. Huang C. P. Zeng D. Z. . (2020). The impact of the sense of life meaning on the quality of life in patients with depressive disorders. Chin. J. Health Psychol. 28, 173–176.

[ref56] Yang D. D. (2024). The effects of martial arts routines intervention on the mental health of high school students: the chain mediating roles of sense of life meaning and psychological resilience. J. Jilin Sport. Univ. 40, 23–30.

[ref57] Zhang Z. Chen W. Li D. (2021). The relationship between physical activity and negative emotions in a large sample of Chinese college students: the mediating role of self-esteem. Front. Psychol. 12:666700. doi: 10.3389/fpsyg.2021.666700

[ref58] Zhang R. X. Lai Q. Z. Tang S. Y. Xiao R. (2018). The moderating and mediating effects of the sense of life meaning on family economic status and depression in university students. Mod. Prev. Med. 45, 1631–1634.

[ref59] Zhao Y. Wang Y. Q. Wang J. Jiang M. M. Wang J. Jin Y. L. (2021). The current situation and correlates of self-injurious behavior, depression, and anxiety among university students. Chin. J. Sch. Health 42, 92–95.

[ref60] Zhou H. Long L. R. (2004). Statistical tests and control methods for common method bias. Adv. Psychol. Sci. 12, 942–950.

